# A Case Report On Bismuth-Induced Encephalopathy Secondary To Pepto-Bismol

**DOI:** 10.51894/001c.164580

**Published:** 2026-07-31

**Authors:** Nathaniel G. Blanchard, Monica Sanjuan, Nathaniel Z. Wang, Sheila C. Lally

**Affiliations:** 1 Henry Ford - Providence

## Introduction

Bismuth subsalicylate is a widely available over-the-counter medication commonly used for gastrointestinal symptoms. Although generally considered safe, prolonged use can result in systemic toxicity, including severe neurological manifestations that are often underrecognized.

## Case Description

We report the case of a 77-year-old male patient with chronic lymphocytic colitis leading to more than two years of daily bismuth subsalicylate use, whose clinical course was marked by progressive encephalopathy, proximal muscle weakness, tremor, and gait instability. The persistence of these symptoms prompted multiple hospitalizations and comprehensive but non-diagnostic evaluations, culminating in a provisional diagnosis of parkinsonism. The patient’s initial symptoms were subtle and sporadic, but within nine months progressed to regular episodes of proximal muscle weakness, fatigue, and periods of impaired balance; all of which significantly affected the patient’s daily activities. Following each episode, his symptoms demonstrated transient improvement; however, six months later, he experienced a marked decline, displaying pronounced tremors, spasticity, and an inability to stand. He was admitted to the hospital for extensive testing without significant findings. During his hospitalization, discontinuation of Pepto-Bismol resulted in stabilization of his symptoms. He subsequently completed a brief course of rehabilitation and was discharged home. At that time, neither the patient nor his providers recognized a potential association between his neurologic manifestations and bismuth use. Although his symptoms improved substantially, he experienced recurrence two months later, including dysphagia and neurogenic incontinence. The dysphagia culminated in an aspiration event requiring emergency evaluation. Upon disclosure that he had resumed Pepto-Bismol following discharge, a consulting gastroenterologist raised concern for bismuth toxicity as a potential etiology of his chronic encephalopathy-like presentation. Serum testing revealed markedly elevated bismuth levels (300 μg/L). The Pepto-Bismol was immediately discontinued, which resulted in normalization of serum bismuth levels and gradual clinical improvement over several months.

## Discussion

This case underscores the diagnostic challenges associated with bismuth-induced neurotoxicity and emphasizes the critical importance of diligent medication reconciliation, including the careful assessment of ostensibly benign over-the-counter agents. The authors aim to increase clinician awareness for this condition, as prompt recognition and discontinuation of bismuth-containing products can lead to substantial neurological recovery and prevent unnecessary diagnostic interventions.

